# Relative Cation-Anion Diffusion in Alkyltriethylammonium-Based Ionic Liquids

**DOI:** 10.3390/ijms23115994

**Published:** 2022-05-26

**Authors:** Danuta Kruk, Elżbieta Masiewicz, Karol Kołodziejski, Roksana Markiewicz, Stefan Jurga

**Affiliations:** 1 Department of Physics and Biophysics, University of Warmia & Mazury in Olsztyn, Oczapowskiego 4, 10-719 Olsztyn, Poland; elzbieta.masiewicz@uwm.edu.pl (E.M.); karol.kolodziejski@uwm.edu.pl (K.K.); 2 NanoBioMedical Centre, Adam Mickiewicz University, Wszechnicy Piastowskiej 3, 61-614 Poznan, Poland; roksana.markiewicz@amu.edu.pl (R.M.); stjurga@amu.edu.pl (S.J.)

**Keywords:** ionic liquids, relaxation, dynamics, diffusion, nuclear magnetic resonance, correlation effects

## Abstract

^19^F Nuclear Magnetic Resonance spin-lattice relaxation experiments have been performed for a series of ionic liquids including the same anion, bis(trifluoromethanesulfonyl)imide, and cations with alkyl chains of different lengths: triethylhexylammonium, triethyloctylammonium_,_ decyltriethylammonium, dodecyltriethylammonium, decyltriethylammonium, and hexadecyltriethylammonium. The experiments have been carried out in a frequency range of 10 kHz to 10 MHz versus temperature. A thorough analysis of the relaxation data has led to the determination of the cation–anion as a relative translation diffusion coefficient. The diffusion coefficients have been compared with the corresponding cation–cation and anion–anion diffusion coefficients, revealing a correlation in the relative translation movement of the anion and the triethylhexylammonium, triethyloctylammonium, decyltriethylammonium, and dodecyltriethylammonium cations, whereas the relative translation diffusion between the anion and the cations with the longer alkyl chains, decyltriethylammonium and hexadecyltriethylammonium, remains rather uncorrelated (correlated to a much lesser extent).

## 1. Introduction

Mechanisms concerning the translation diffusion of ionic liquids raise a great deal of interest in the field of fundamental science, as well as in terms of its use in applications. In both cases, one of the most important questions concerns the correlation effects in the ionic movement. One wonders to what extent the translation displacement of one ion (cation or anion) affects the movement of other ions (of the same and of the opposite sign) as a result of their mutual interactions. 

It is very difficult to address this subject experimentally. Nuclear Magnetic Resonance (NMR) methods are able to give insight into ionic motion. NMR diffusometry probes the translational movement of individual ions in a magnetic field gradient [1,2]. For ionic liquids composed of ^1^H containing cations and ^19^F containing anions, one can obtain translation diffusion coefficients of the cation and the anion, respectively. The diffusion coefficients describe self-diffusion of the ions. This information is highly valuable, but it does not give insight into motional correlation. NMR relaxometry probes relative translational dynamics [3,4,5,6,7,8,9,10,11,12,13]. “Classical” NMR experiments are carried out in a single, high magnetic field. The Fast Field Cycling technology [14,15] applied in NMR relaxometry enables NMR relaxation experiments to be performed in a very broad range of magnetic fields (and, hence, resonance frequencies are proportional to the magnetic field)—in the present work they are performed in a range of 10 kHz to 10 MHz (referring to ^1^H resonance frequency). The principle of the relaxation process is as follows. ^1^H (^19^F) nuclei can occupy two energy levels in an external magnetic field, which are associated with parallel and anti-parallel orientations of the magnetic moments of the nuclei with respect to the direction of the magnetic field. The molecular or ionic system gains magnetization as a result of the difference in the populations of the two energy levels, as according to the Boltzmann distribution, the population of the lower energy level is higher. When the magnetic field is altered, the populations of the two energy levels change, thus reaching a new equilibrium (according to the Boltzmann distribution). This process is referred to as spin-lattice relaxation. The repopulation of the energy levels is observed as being an evolution of magnetization over time. In most cases, there is a single-exponential evolution, and the characteristic time constant of the magnetization dependence on time is referred to as the spin-lattice relaxation time (and its reciprocal value is called the spin-lattice relaxation rate). The repopulation of the energy levels (i.e., the relaxation process) requires exchanging energy with surrounding molecules or ions, which is referred to as a lattice. The exchange occurs via magnetic dipole–dipole interactions with neighbouring NMR active nuclei. The interactions fluctuate in time as a result of the molecular or ionic motion. The strength of the interactions, as well as the timescale of the motion and its mechanism, determine the relaxation rates.

According to spin relaxation theory [16,17,18], at a given resonance frequency, the dominating contribution to the spin-lattice relaxation is associated with a dynamic process occurring on a timescale matching the reciprocal frequency. This statement is somewhat oversimplified as it does not account for the corresponding interaction strengths; however it captures the essential feature: with increasing frequencies, one obtains progressively faster dynamics. Consequently, NMR relaxometry gives access to molecular motion on the timescale from ms to ns, whereas “classical” NMR relaxation experiments give access to a much faster motion.

Dipole–dipole interactions leading to the relaxation process can be of inter-molecular (inter-ionic) or intra-molecular (intra-ionic) origin. The inter-molecular (inter-ionic) interactions fluctuate in time due to the relative translation diffusion of the interacting species, whereas the intra-molecular (intra-ionic) ones are modulated by rotational dynamics. Consequently, NMR relaxometry is a very valuable source of information in terms of understanding dynamic processes in condensed matter. This method has extensively been exploited for investigating the translational and rotational dynamics of molecular liquids; significantly fewer studies have been performed for ionic liquids. The applications of NMR relaxometry for ionic liquids in bulk [4,6,7,8,10] and confinement [8,12,13] have given a very valuable insight into the dynamics of cations and anions, especially for liquids composed of ^1^H containing cations and ^19^F containing anions.

In this work, we exploit NMR relaxometry to enquire into the relative cation–anion dynamics in a series of ionic liquids composed of the same (^19^F containing) anion, bis(trifluoromethanesulfonyl)imide, [TFSI], and (^1^H containing) cations that differ with respect to the length of the alkyl chain: triethylhexylammonium bis(trifluoromethanesulfonyl)imide triethyloctylammonium ([TEA–C6] [TFSI])—C_14_H_28_F_6_N_2_O_4_S_2_, bis(trifluoromethanesulfonyl)imide ([TEA–C8] [TFSI])—C_16_H_32_F_6_N_2_O_2_S_2_, dodecyltriethylammonium bis(trifluoromethanesulfonyl)imide ([TEA–C12] [TFSI])—C_20_H_40_F_6_N_2_O_2_S_2_, and hexadecyltriethylammonium bis(trifluoromethanesulfonyl)imide ([TEA–C16] [TFSI])—C_24_H_48_F_6_N_2_O_2_S_2_. The inspiration for these studies is our recent work [7], which focused on the dynamical properties of butyltriethylammonium bis(trifluoromethanesulfonyl)imide ([TEA–C4] [TFSI])—C_12_H_24_F_6_N_2_O_2_S_2_, especially the ^19^F relaxation experiments which were performed for [TEA–C4] [TFSI]. The ^19^F spin-lattice relaxation rates for [TEA–C4] [TFSI] stem from two main relaxation contributions that are associated with the anion—cation (^19^F-^1^H) and anion—anion (^19^F-^19^F) dipole–dipole interactions, respectively. The ^19^F-^1^H dipole–dipole interactions fluctuate in time as a result of the relative cation–anion translation diffusion, whereas the ^19^F-^19^F dipole–dipole coupling is modulated by the relative anion–anion translation diffusion; however, as a result of the ratio between the numbers of ^1^H and ^19^F nuclei in [TEA–C4] [TFSI] (this subject is explained in detail in Section 2), the overall ^19^F spin-lattice relaxation rate is dominated by the ^19^F-^1^H relaxation contribution. Moreover, one can expect (and this has proven to be true) that by increasing the length of the alkyl chain (and, hence, increasing the ratio between the number of ^1^H and ^19^F nuclei), the ^19^F-^19^F relaxation contribution will become negligible. This creates an extraordinary opportunity to enquire into the relative cation–anion translation diffusion. One should stress at this stage, that with separate NMR diffusometry probes, the cation and the anion self-diffuse, but this does not give any insight into the relative cation–anion motion. To our knowledge, this is the first example of a direct, experimental determination of the relative cation–anion translation diffusion coefficient for ionic liquids. Moreover, the ^19^F spin-lattice relaxation experiments for the series of ionic liquids, complemented by the results for [TEA–C4] [TFSI] [4], give insight into how the structure of the cation affects the relative cation–anion movement. 

In cases of isotropic (three-dimensional) translation diffusion (characteristic of liquids in bulk), at low frequencies, one observes a linear dependence of the spin-lattice relaxation rate at the squared root of the resonance frequency [19,20,21,22,23]. In cases of inter-molecular (inter-ionic) interactions, the relaxation rate is dominated by a relaxation contribution originating from dipole–dipole interactions between one kind of NMR active nuclei (^1^H-^1^H or ^19^F-^19^F). Moreover, the translation diffusion coefficient can be determined from the low frequency slope of the relaxation rate versus the squared root of the frequency [7,23]. In this work, a corresponding expression has been described and applied for the case of ^1^H-^19^F dipole–dipole interactions, enabling a straightforward determination of the cation–anion relative translation diffusion coefficient.

## 2. Results

**Figure 1:**
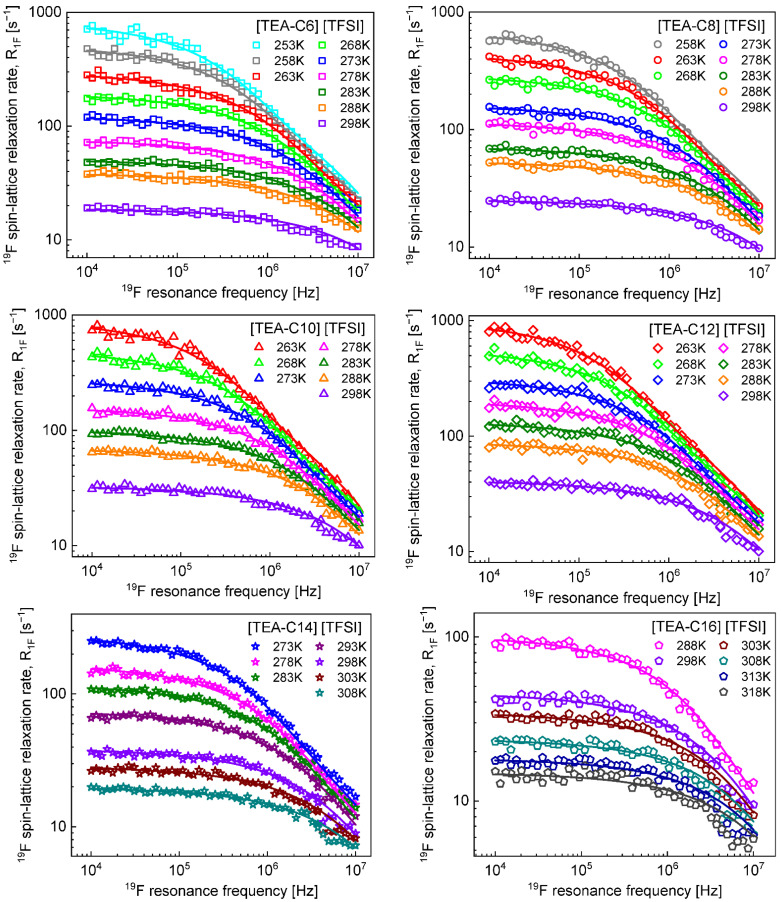
^19^F spin-lattice relaxation data for a series of ionic liquids: [TEA–C6] [TFSI], [TEA–C8] [TFSI], [TEA–C10] [TFSI], [TEA–C12] [TFSI], [TEA–C14] [TFSI], and [TEA–C16] [TFSI]. Solid lines—fits in terms of Equation (1).

Figure 1 shows ^19^F spin-lattice relaxation data for the series of ionic liquids: [TEA–C6] [TFSI], [TEA–C8] [TFSI], [TEA–C10] [TFSI], [TEA–C12] [TFSI], [TEA–C14] [TFSI], and [TEA–C16] [TFSI], presented in Table 1. The data have been analysed in terms of Equation (1) of Section 4.

The obtained cation–anion relative translation diffusion coefficients, $D_{trans}^{CA}$, and the cation–anion distance of closest approach, $d_{CA}$, are listed in Table 2.

**Figure 2:**
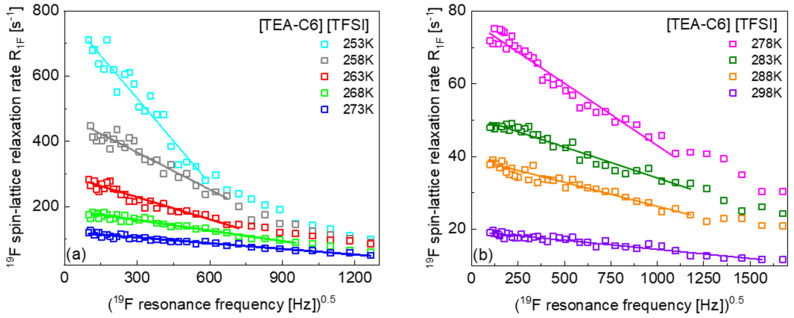
^19^F spin-lattice relaxation data for [TEA–C6] [TFSI] versus the squared root of the resonance frequency; solid lines represent linear fits in the low frequency range.

**Figure A1:**
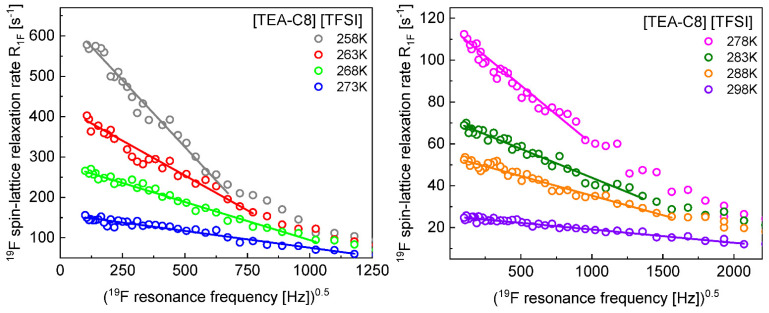
^19^F spin-lattice relaxation data for [TEA–C8] [TFSI] versus the squared root of the resonance frequency; solid lines represent linear fits in the low frequency range.

**Figure A2:**
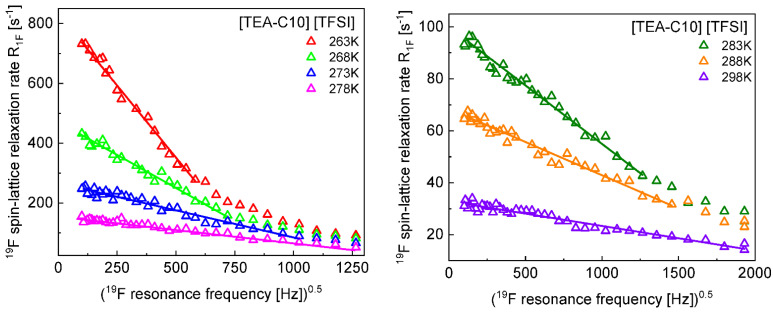
^19^F spin-lattice relaxation data for [TEA–C10] [TFSI] versus the squared root of the resonance frequency; solid lines represent linear fits in the low frequency range.

**Figure A3:**
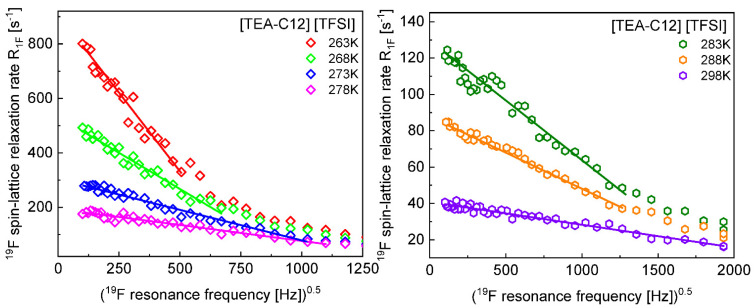
^19^F spin-lattice relaxation data for [TEA–C12] [TFSI] versus the squared root of the resonance frequency; solid lines represent linear fits in the low frequency range.

**Figure A4:**
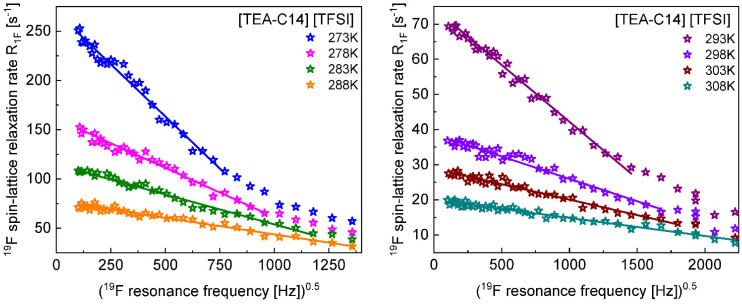
^19^F spin-lattice relaxation data for [TEA–C14] [TFSI] versus the squared root of the resonance frequency; solid lines represent linear fits in the low frequency range.

**Figure A5:**
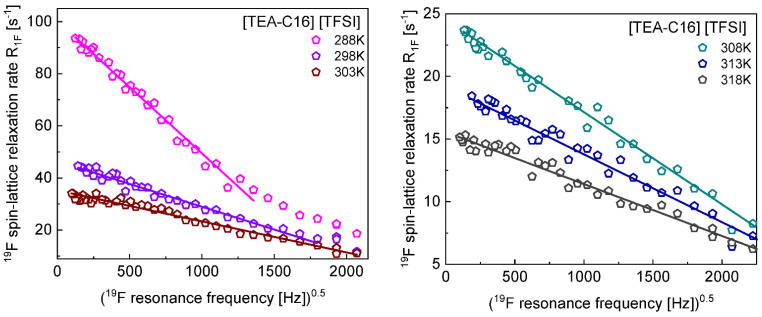
^19^F spin-lattice relaxation data for [TEA–C14] [TFSI] versus the squared root of the resonance frequency; solid lines represent linear fits in the low frequency range.

In addition, the relative translation diffusion coefficients have been obtained from the low frequency slope of the relaxation rates plotted, versus the squared root of the resonance frequency, in terms of Equation (3). Figure 2a,b shows the relaxation data for [TEA–C6] [TFSI] in this representation. The ^19^F spin-lattice relaxation rates for [TEA–C8] [TFSI], [TEA–C10] [TFSI], [TEA–C12] [TFSI], [TEA–C14] [TFSI], and [TEA–C16] [TFSI] plotted, versus the squared root of the resonance frequency, accompanied by the corresponding low frequency fits, are shown in Appendix B (Figure A1, Figure A2, Figure A3, Figure A4 and Figure A5). The cation–anion translation diffusion coefficients obtained from the low frequency slopes are included in Table 2. They are in very good agreement with those obtained from the fits of the relaxation data in terms of Equation (3).

**Figure 3:**
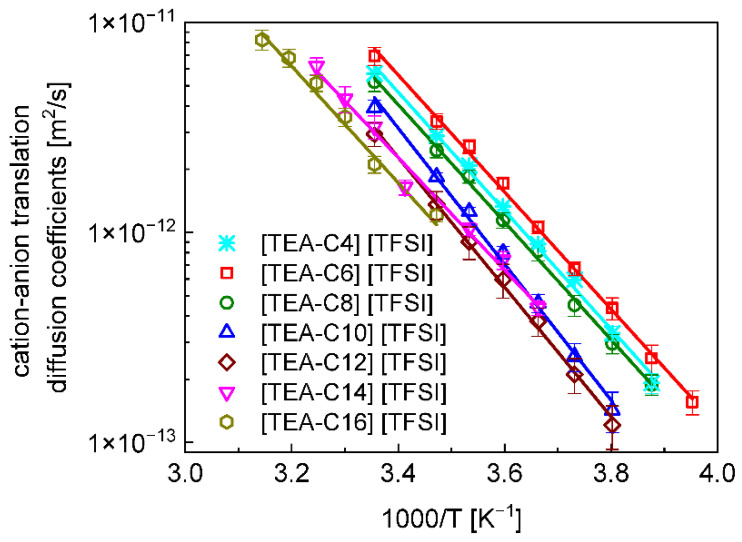
Relative cation–anion translation diffusion coefficients, $D_{trans}^{CA}$, for [TEA–C4] [TFSI], [TEA–C6] [TFSI], [TEA–C8] [TFSI], [TEA–C10] [TFSI], [TEA–C12] [TFSI], [TEA–C14] [TFSI], and [TEA–C16] [TFSI]. The data for [TEA–C4] [TFSI] are taken from [4]. Solid lines—fits according to the Arrhenius law: $D_{trans}^{CA}=D_{trans,0}^{CA}exp\left(-\frac{E_{A}}{RT}\right)$, where $E_{A}$ denotes the activation energy.

The translation diffusion coefficients, obtained from the full fits, are plotted in Figure 3 versus the reciprocal temperature. The data are complemented by the relative cation–anion translation diffusion coefficients for [TEA–C4] [TFSI], which are taken from [4].

The activation energies obtained from the Arrhenius fits are: [TEA–C4] [TFSI]—(23.4 ± 0.6) kJ/mol [7], [TEA–C6] [TFSI]—(23.2 ± 0.4) kJ/mol, [TEA–C8] [TFSI]—(23.3 ± 0.5) kJ/mol, [TEA–C10] [TFSI]—(26.9 ± 0.7) kJ/mol, [TEA–C12] [TFSI]—(25.7 ± 0.6) kJ/mol, [TEA–C14] [TFSI]—(23.4 ± 0.6) kJ/mol and [TEA–C16] [TFSI]—(19.4 ± 0.5) kJ/mol. 

**Figure 4:**
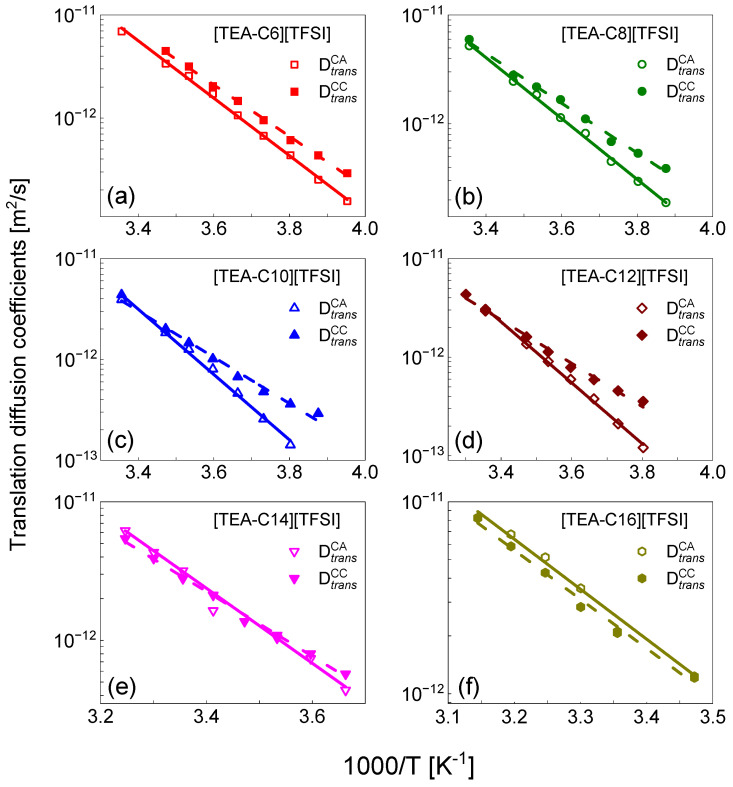
Relative cation–anion translation diffusion coefficients, $D_{trans}^{CA}$, and cation–cation translation diffusion coefficients, $D_{trans}^{CC}$, for (**a**) [TEA–C6] [TFSI], (**b**) [TEA–C8] [TFSI], (**c**) [TEA–C10] [TFSI], (**d**) [TEA–C12] [TFSI], (**e**) [TEA–C14] [TFSI], and (**f**) [TEA–C16] [TFSI] (the $D_{trans}^{CC}$ values are taken from [6]). The solid lines fit according to the Arrhenius law for $D_{trans}^{CA}$, and the dashed lines fit according to the Arrhenius law for $D_{trans}^{CC}$ (taken from [6]).

It is of interest to compare the relative cation–anion translation diffusion coefficients with the relative cation–cation diffusion coefficients for [TEA–C6] [TFSI], [TEA–C8] [TFSI], [TEA–C10] [TFSI], [TEA–C12] [TFSI], [TEA–C14] [TFSI], and [TEA–C16] [TFSI] which were obtained in [6]. The comparison is shown in Figure 4.

The activation energies of the cation–cation translation dynamics are [9]: (20.8 ± 0.4) kJ/(mol × K) for [TEA–C6] [TFSI], (19.0 ± 0.6) kJ/(mol × K) for [TEA–C8] [TFSI], (19.0 ± 0.9) kJ/(mol × K) for [TEA–C10] [TFSI], (18.3 ± 0.7) kJ/(mol × K) for [TEA–C12], (19.5 ± 0.5) kJ/(mol × K) for [TEA–C14] [TFSI], (21.3 ± 1.0) kJ/(mol × K) for [TEA–C16] [TFSI]), and (23.4 ± 0.6) kJ/(mol × K) for [TEA–C4] [TFSI] [4].

## 3. Discussion

The ^19^F spin-lattice relaxation studies have revealed that the relative cation–anion translation diffusion coefficients for the series of ionic liquids [TEA–C6] [TFSI], [TEA–C8] [TFSI], [TEA–C10] [TFSI], [TEA–C12] [TFSI], [TEA–C14] [TFSI], and [TEA–C16] [TFSI], including [TEA–C4] [TFSI] [7], range between 10^−11^ m^2^/s and 10^−13^ m^2^/s in the considered temperature range. Figure 3 shows that the fastest cation–anion diffusion is observed for [TEA–C6] [TFSI], whereas for [TEA–C4] [TFSI] and [TEA–C8] [TFSI], the translation diffusion coefficients are very similar and somewhat lower than for [TEA–C6] [TFSI]. Although the differences are not large, they show that the cation–anion translation diffusion coefficients do not change monotonically with the length of the alkyl chain. One should note the identical activation energies for [TEA–C4] [TFSI] [7], TEA–C6] [TFSI], and [TEA–C8] [TFSI] ((23.4 ± 0.6) kJ/mol, (23.2 ± 0.4) kJ/mol and (23.3 ± 0.5) kJ/mol for [TEA–C4] [TFSI], [TEA–C6] [TFSI] and [TEA–C8] [TFSI], respectively). It is worth noting at this stage that although the activation energy of the cation–cation translation motion for [TEA–C4] [TFSI], (23.4 ± 0.6) kJ/(mol × K) [4] is the same (within the uncertainty limits) as the cation–anion diffusion for [TEA–C6] [TFSI] and [TEA–C8] [TFSI], the activation energy for the cation–cation motion is lower ((20.8 ± 0.4) kJ/(mol × K) for [TEA–C6] [TFSI] and (19.0 ± 0.6) kJ/(mol × K) for [TEA–C8] [TFSI] [6]) than that of the cation–anion diffusion. Following this, with the increasing the length of the alkyl chain, the cation–anion translation diffusion for [TEA–C10] [TFSI] is slower than for the counterparts with shorter alkyl chains (Figure 3). Moreover, the activation energy, (26.9 ± 0.7) kJ/mol, is much higher compared with that of the cation–cation motion, (19.0 ± 0.9) kJ/(mol × K) [6]. With the alkyl chain length increasing further ([TEA–C12] [TFSI]), the cation–anion diffusion becomes slower, and the activation energy for the cation–anion motion ((25.7 ± 0.6) kJ/mol) still remains larger than that of the cation–cation motion, (18.3 ± 0.7) kJ/(mol × K) [6]. This trend changes for [TEA–C14] [TFSI]; at higher temperatures, the cation–anion diffusion coefficient converges to the one for [TEA–C12] [TFSI], whereas at lower temperatures, it reaches values similar to those for [TEA–C10] [TFSI]. The activation energy is, in fact, the same as for [TEA–C4] [TFSI], [TEA–C6] [TFSI], and [TEA–C10] [TFSI], and higher than that of the cation–cation motion ((19.5 ± 0.5) kJ/(mol × K) [9]). Eventually, for [TEA–C16] [TFSI], the cation–anion motion slows down further as the activation energy for the cation–anion diffusion ((19.4 ± 0.5) kJ/mol) is lower than that of the cation–cation motion ((21.3 ± 1.0) kJ/(mol × K) [6]).

In [6] the relative cation–cation, anion–anion, and cation–anion translational diffusion coefficients, [TEA–C14] [TFSI] was determined. It has been shown that the cation–cation diffusion is considerably slower than the anion–anion relative translation movement. This was expected, taking into account the size of the cation and anion. It is, however, worth to noting that at lower temperatures, the diffusion coefficients tend to converge. As NMR relaxometry probes the relative translation diffusion coefficients, in [6], the cation–cation diffusion coefficients have been compared with the cation self-diffusion coefficients, showing that the relative diffusion coefficients are somewhat lower than twice the self-diffusion ones. This has been attributed to the correlation effects in the cation–cation relative movement. It might be worth saying at this stage, that for uncorrelated dynamics, the relative diffusion coefficient is given as a sum of self-diffusion coefficients of the interacting species (so twice the self-diffusion coefficient in case they are identical). The same observation has been made for the liquids [TEA–C6] [TFSI], [TEA–C8] [TFSI], [TEA–C10] [TFSI], [TEA–C12] [TFSI], [TEA–C14] [TFSI], and [TEA–C16] [TFSI] in [6,24]—the relative cation–cation translation diffusion coefficients are lower than twice the corresponding self-diffusion ones. As the anion diffusion is faster than the cation diffusion (although the anion–anion diffusion coefficients in [TEA–C6] [TFSI], [TEA–C8] [TFSI], [TEA–C10] [TFSI], [TEA–C12] [TFSI], [TEA–C14] [TFSI], and [TEA–C16] [TFSI] are not determined because, as already explained, the ^19^F-^19^F relaxation contribution to the ^19^F relaxation becomes negligible as the length of the alkyl chain increases, thus, this statement is justified), the relative translation diffusion coefficient for uncorrelated cation–anion motion cannot be lower than the cation–cation diffusion coefficient. One can clearly see from Figure 4, that for [TEA–C6] [TFSI], [TEA–C8] [TFSI], [TEA–C10] [TFSI], and [TEA–C12] [TFSI], the relative cation–anion translation diffusion coefficient, $D_{trans}^{CA}$, is lower (the diffusion is slower) than the cation–cation diffusion coefficient $D_{trans}^{CC}$ (analogous relationship that has been observed for [TEA–C14] [TFSI] [4]), and this effect increases with decreasing temperatures. This is a strong indication that the cation–anion translation movement for [TEA–C6] [TFSI], [TEA–C8] [TFSI], [TEA–C10] [TFSI], and [TEA–C12] [TFSI] is correlated, and the effects are more pronounced with decreasing temperatures. For [TEA–C14] [TFSI] and [TEA–C16] [TFSI], this effect is not observed. Taking into account that experimental studies of correlated ionic dynamics are rare and difficult, this finding gives a unique insight into the correlation of the relative cation–anion movement in ionic liquids.

## 4. Materials and Methods

^19^F spin-lattice relaxation measurements have been performed for [TEA–C6] [TFSI], [TEA–C8] [TFSI], [TEA–C10] [TFSI], [TEA–C12] [TFSI], [TEA–C14] [TFSI], and [TEA–C16] [TFSI] in the frequency range from 10 kHz to 10 MHz versus temperature, using a NMR relaxometer, produced by Stelar s.r.l. (Mede (PV), Italy). The compounds were placed in NMR glass tubes at diameters of 7 nm and sealed. The liquids were dried for 48 h in a vacuum desiccator before the experiment. The temperature was controlled with an accuracy of 0.5 K. For each resonance frequency, 32 magnetization values have been recorded, versus time in a logarithmic time scale. The relaxation processes were single-exponential for all temperatures in the whole frequency range for all liquids. Examples of magnetization curves (^19^F magnetization versus time) are shown in the Supplementary Material.

Table 1 includes the list of the liquids with their molecular mass and density. Moreover, the numbers of ^1^H and ^19^F nuclei per unit volume have been provided. The number of ^1^H nuclei (hydrogen atoms) per unit volume has been obtained from the relationship: $N_{H}=\frac{n_{H}N_{A}\varrho }{M}$, where $n_{H}$ denotes the number of hydrogen atoms per cation, $N_{A}$ is the Avogadro number, ϱ denotes density of the ionic liquid, and M is its molecular mass. The $N_{F}$ number is given as: $N_{F}=\frac{n_{F}}{n_{H}}N_{H}$, where $n_{F}$ denotes the number of fluorine atoms per anion.

The synthesis procedure of the series of ionic liquids has been described in detail in [24].

The applied theoretical model is presented below.

^19^F relaxation processes in ionic liquids composed of ^1^H containing cations and ^19^F containing anions stem from three relaxation channels: ^1^H-^19^F magnetic dipole–dipole interactions modulated by relative cation–anion translation diffusion, ^19^F-^19^F dipole–dipole interactions between anions (inter-anionic), modulated by a relative anion–anion translation motion, and ^19^F-^19^F dipole–dipole interactions within the anion (intra-anionic, provided the anion includes more than one ^19^F nuclei), modulated by the rotational dynamics of the anion. For the investigated series of ionic liquids, the ^19^F spin-lattice relaxation rate, $R_{1F}\left(\omega_{F}\right)$ ($\omega_{F}\;$denotes ^19^F resonance frequency in angular frequency units), is dominated by the relaxation contribution originating from the ^1^H-^19^F cation–anion dipole–dipole interactions. Consequently, the relaxation rate, $R_{1F}\left(\omega_{F}\right)$, is given as [4,7]:

(1) $$\begin{array}{ll} R_{1F}^{}\left(\omega_{F}\right)=R_{1F}^{HF} & \left(\omega_{F}\right)\;\\ & =\frac{36}{5}{\left(\frac{\mu_{0}}{4\pi }\gamma_{H}\gamma_{F}\hbar \right)}^{2}\frac{1}{d_{CA}^{3}}N_{H}\int_{0}^{\infty }\frac{u^{4}}{81+9u^{2}-2u^{4}+u^{6}}[\frac{\tau_{trans}^{CA}}{u^{4}+{\left(\left(\omega_{H}-\omega_{F}\right)\tau_{trans}^{CA}\right)}^{2}}\\ & +\frac{3\tau_{trans}^{CA}}{u^{4}+{\left(\omega_{F}\tau_{trans}^{CA}\right)}^{2}}+\frac{6\tau_{trans}^{CA}}{u^{4}+{\left(\left(\omega_{H}+\omega_{F}\right)\tau_{trans}^{CA}\right)}^{2}}] \end{array}$$

where the correlation time $\tau_{trans}^{CA}$ is defined as: $\tau_{trans}^{CA}=\frac{d_{CA}^{2}}{D_{trans}^{CA}}$ ($D_{trans}^{CA}\;$ denotes a relative cation–anion translation diffusion coefficient, whereas $d_{CA}$ is the cation–anion distance of closest approach), $N_{H}$ denotes the number of ^1^H nuclei per unit volume, whereas $\gamma_{F}$ and $\gamma_{H}$ are ^19^F and ^1^H gyromagnetic factors, respectively; $\omega_{H}$ denotes the ^1^H resonance frequency in angular frequency units. The relaxation contribution originating from anion–anion and ^19^F-^19^F dipole–dipole interactions is proportional to the number of ^19^F nuclei per unit volume, $N_{F}$. This explains why this relaxation contribution is much lower than that originating from ^1^H-^19^F interactions—the $N_{H}$ number is much larger than $N_{F}$ (Table 1). It has been shown already, that for [TEA–C4] [TFSI], the anion–anion relaxation contribution to ^19^F spin-lattice relaxation is much lower than the cation–anion term [4]. By increasing the chain length, and given the $N_{H}$ number compared with $N_{F}$, the ^19^F-^19^F relaxation contribution becomes negligible. As far as the intra-anionic relaxation contribution is concerned, the relaxation rates are low because of the fast rotational dynamics of the TFSI cations, which has already been shown in [4]. This creates a unique opportunity to solely probe the relative cation–anion translation movement.

The translational spectral density:

(2) $$J_{trans}\left(\omega \right)=\frac{72}{5}\frac{1}{d^{3}}N\int_{0}^{\infty }\frac{u^{2}}{81+9u^{2}-2u^{4}+u^{6}}\frac{u^{2}\tau_{trans}}{u^{4}+{\left(\omega \tau_{trans}\right)}^{2}}du$$

(here, we used the symbols $\tau_{trans\;}$, d , and N, which demonstrate the general meaning of this formulae) can be expanded into the Taylor series in the range of ωτ<1. The expansion takes the form [20,21,23]:

(3) $$J_{trans}\left(\omega \right)≅a-b\sqrt{\omega },\;b=\frac{2^{3/2}\pi }{45D_{trans}^{3/2}}$$

where $D_{trans}$ denotes a relative translation diffusion coefficient; a is a frequency independent term. This implies that for isotropic translational diffusion, one observes a linear dependence of the spin-lattice relaxation rates on the square root of the resonance frequency. Moreover, from the low frequency slope, one can straightforwardly determine the translation diffusion coefficient [7,23]. An expression derived on this basis has been used for molecular and ionic liquids when the dominating relaxation contribution originates from ^1^H-^1^H dipole–dipole interactions. Combining Equation (3) with Equation (1), one can determine a relationship between the low frequency slope, B, and the relative cation–anion translation diffusion coefficient for the present case:

(4) $$B=\frac{2^{1/2}\pi }{45}{\left(\frac{\mu_{0}}{4\pi }\gamma_{H}\gamma_{F}\hbar \right)}^{2}\left[{\left(\frac{\omega_{H}-\omega_{F}}{\omega_{F}}\right)}^{1/2}+3+6{\left(\frac{\omega_{H}+\omega_{F}}{\omega_{F}}\right)}^{1/2}\right]N_{H}{\left(D_{trans}^{CA}\right)}^{-3/2},\;$$

Taking into account that $\omega_{H}≅\omega_{F}$, one can simplify the relationship to form:

(5) $$B=\frac{2^{1/2}\pi \left(1+2^{3/2}\right)}{15}{\left(\frac{\mu_{0}\gamma_{H}^{}\gamma_{F}\hbar }{4\pi }\right)}^{2}N_{H}{\left(D_{trans}^{CA}\right)}^{-3/2}$$

A detailed derivation of the expression can be found in Appendix A.

## 5. Conclusions

It has turned out that the ^19^F spin-lattice relaxation data for [TEA–C6] [TFSI], [TEA–C8] [TFSI], [TEA–C10] [TFSI], [TEA–C12] [TFSI], [TEA–C14] [TFSI], and [TEA–C16] [TFSI] can be fully attributed, in the frequency range from 10kHz to 10MHz, to the cation–anion ^1^H-^19^F dipole–dipole interactions. Consequently, this paved the way for the determination of the relative cation–anion translation diffusion coefficients. In addition to the analysis of the relaxation data in terms of a full fit, a formula allowing the straightforward determination of the relative translation diffusion coefficient, from the slow, low frequency of the relaxation rates plotted, versus the squared root of the resonance frequency, has been ascertained. The values of the diffusion coefficients, obtained in both ways, are in good agreement with one another. The cation–anion diffusion coefficients have been compared with the corresponding values of the cation–cation diffusion coefficients taken from [9]. With the knowledge that the anion diffusion is not slower than the translation motion of the cations [9], it has been concluded that for [TEA–C6] [TFSI], [TEA–C8] [TFSI], [TEA–C10] [TFSI], and [TEA–C12] [TFSI], the cation–anion translation movement shows correlation effects increasing with decreasing temperatures. Such effects have not been observed for the ionic liquids, including cations with longer alkyl chains, [TEA–C14] [TFSI] and [TEA–C16] [TFSI].

## Figures and Tables

**Table 1:** Properties of the series of ionic liquids.

Name; Abbreviation	Chemical Formula	Molecular Mass [g/mol]	Density [g/cm^3^]	$N_{H}\;[m^{-3}]$	$N_{F}\;[m^{-3}]$
Triethylhexylammonium bis(trifluoromethanesulfonyl)imide; [TEA–C6] [TFSI]	C_14_H_28_F_6_N_2_O_4_S_2_	466.50	1.29	4.66 × 10^28^	9.99 × 10^28^
Triethyloctylammonium bis(trifluoromethanesulfonyl)imide; [TEA–C8] [TFSI]	C_16_H_32_F_6_N_2_O_4_S_2_	494.56	1.25	4.87 × 10^28^	9.13 × 10^27^
Decyltriethylammonium bis(trifluoromethanesulfonyl)imide; [TEA–C10] [TFSI]	C_18_H_36_F_6_N_2_O_4_S_2_	522.61	1.21	5.02 × 10^28^	8.36 × 10^27^
Dodecyltriethylammonium bis(trifluoromethanesulfonyl)imide; [TEA–C12] [TFSI]	C_20_H_40_F_6_N_2_O_4_S_2_	550.66	1.17	5.12 × 10^28^	7.94 × 10^28^
Triethyltetradecylammonium bis(trifluoromethanesulfonyl)imide; [TEA–C14] [TFSI]	C_22_H_44_F_6_N_2_O_4_S_2_	578.72	1.16	5.31 × 10^28^	7.24 × 10^27^
Hexadecyltriethylammonium bis(trifluoromethanesulfonyl)imide; [TEA–C16] [TFSI]	C_24_H_48_F_6_N_2_O_4_S_2_	606.77	1.13	5.38 × 10^28^	6.73 × 10^27^

**Table 2:** Properties of the series of ionic liquids.

Temp. [K]	$D_{trans}^{CA}\;[m^{2}/s]$	Rel. Error [%]	$\tau_{trans}^{CA}\;[s]$	$D_{trans}^{CA}\;[m^{2}/s],$ Slope
$[\mathrm{TEA}–C6]\;[\mathrm{TFSI}]\;\;d_{CA}=$ 3.36 Å				
253	1.56 × 10^−13^	13.1	1.76 × 10^−12^	1.85 × 10^−13^
258	2.52 × 10^−13^	15.3	2.84 × 10^−12^	2.38 × 10^−13^
263	4.36 × 10^−13^	11.9	4.92 × 10^−12^	4.76 × 10^−13^
268	6.72 × 10^−13^	7.7	7.58 × 10^−12^	6.86 × 10^−13^
273	1.06 × 10^−12^	6.9	1.20 × 10^−11^	1.18 × 10^−12^
278	1.72 × 10^−12^	6.5	1.94 × 10^−11^	1.72 × 10^−12^
283	2.57 × 10^−12^	7.3	2.90 × 10^−11^	2.68 × 10^−12^
288	3.38 × 10^−12^	8.3	3.82 × 10^−11^	3.32 × 10^−12^
298	6.92 × 10^−12^	9.8	7.82 × 10^−11^	6.88 × 10^−12^
$[\mathrm{TEA}–C8]\;[\mathrm{TFSI}]\;\;d_{CA}=$ 3.43 Å				
258	1.89 × 10^−13^	11.1	2.22 × 10^−12^	1.84 × 10^−13^
263	2.96 × 10^−13^	10.5	3.48 × 10^−12^	2.58 × 10^−13^
268	4.51 × 10^−13^	11.4	5.30 × 10^−12^	4.56 × 10^−13^
273	8.18 × 10^−13^	10.4	9.62 × 10^−12^	8.58 × 10^−13^
278	1.14 × 10^−12^	8.5	1.34 × 10^−11^	1.26 × 10^−12^
283	1.85 × 10^−12^	7.1	2.18 × 10^−11^	1.92 × 10^−12^
288	2.46 × 10^−12^	7.7	2.90 × 10^−11^	2.46 × 10^−12^
298	5.21 × 10^−12^	10.1	6.14 × 10^−11^	5.26 × 10^−12^
[TEA–$C10]\;[\mathrm{TFSI}]\;\;d_{CA}=$ 3.64 Å				
263	1.42 × 10^−13^	21.7	1.89 × 10^−12^	1.93 × 10^−13^
268	2.57 × 10^−13^	15.3	3.40 × 10^−12^	3.30 × 10^−13^
273	4.61 × 10^−13^	10.1	6.10 × 10^−12^	5.88 × 10^−13^
278	8.03 × 10^−13^	6.7	1.06 × 10^−11^	7.78 × 10^−13^
283	1.26 × 10^−12^	4.6	1.67 × 10^−11^	1.49 × 10^−12^
288	1.84 × 10^−12^	4.7	2.44 × 10^−11^	2.16 × 10^−12^
298	3.92 × 10^−12^	8.7	5.20 × 10^−11^	4.20 × 10^−12^
$[\mathrm{TEA}–C12]\;[\mathrm{TFSI}]\;\;d_{CA}=$ 3.73 Å				
263	1.21 × 10^−13^	15.7	1.68 × 10^−12^	1.74 × 10^−13^
268	2.11 × 10^−13^	12.8	2.94 × 10^−12^	2.88 × 10^−13^
273	3.78 × 10^−13^	10.1	5.26 × 10^−12^	3.72 × 10^−13^
278	5.96 × 10^−13^	12.3	8.30 × 10^−12^	5.94 × 10^−13^
283	9.03 × 10^−13^	11.7	1.26 × 10^−11^	9.16 × 10^−13^
288	1.36 × 10^−12^	10.1	1.89 × 10^−11^	1.36 × 10^−12^
298	2.94 × 10^−12^	8.6	4.10 × 10^−11^	2.95 × 10^−12^
$[\mathrm{TEA}–C14]\;[\mathrm{TFSI}]\;\;d_{CA}=4.52\;$Å				
273	4.37 × 10^−13^	5.1	6.18 × 10^−12^	4.46 × 10^−13^
278	7.39 × 10^−13^	6.5	8.73 × 10^−12^	7.98 × 10^−13^
283	1.04 × 10^−12^	4.5	1.81 × 10^−11^	1.05 × 10^−12^
293	1.64 × 10^−12^	5.5	2.63 × 10^−11^	1.91 × 10^−12^
298	3.18 × 10^−12^	8.6	3.46 × 10^−11^	3.19 × 10^−12^
303	4.32 × 10^−12^	9.5	4.22 × 10^−11^	4.42 × 10^−12^
308	6.19 × 10^−12^	6.2	3.16 × 10^−11^	6.39 × 10^−12^
$[\mathrm{TEA}–C16]\;[\mathrm{TFSI}]\;\;d_{CA}=$ 4.73 Å				
288	1.21 × 10^−12^	7.1	1.35 × 10^−11^	1.25 × 10^−12^
298	2.11 × 10^−12^	9.3	1.91 × 10^−11^	1.74 × 10^−12^
303	3.54 × 10^−12^	9.9	3.96 × 10^−11^	3.45 × 10^−12^
308	5.14 × 10^−12^	8.8	6.05 × 10^−11^	5.28 × 10^−12^
313	6.77 × 10^−12^	9.7	7.57 × 10^−11^	6.78 × 10^−12^
318	8.26 × 10^−12^	11.0	9.24 × 10^−11^	8.27 × 10^−12^

## Data Availability

The data is available from the corresponding Author.

## Supplementary Materials

The following supporting information can be downloaded at: https://www.mdpi.com/article/10.3390/ijms23115994/s1.

## Appendix A

Equation (1) can be rewritten in the form:

(A1) $$R_{1F}^{}\left(\omega_{F}\right)=\frac{1}{2}{\left(\frac{\mu_{0}}{4\pi }\gamma_{H}\gamma_{F}\hbar \right)}^{2}N_{H}\left[J_{trans}\left(\omega_{F}-\omega_{H}\right)+3J_{trans}\left(\omega_{F}\right)+6J_{trans}\left(\omega_{F}+\omega_{H}\right)\right]$$

where the spectral density $J_{trans}\left(\omega \right)$ is defined by Equation (2).

Using Equation (3), one obtains for the individual terms:

(A2) $$\frac{1}{2}{\left(\frac{\mu_{0}}{4\pi }\gamma_{H}\gamma_{F}\hbar \right)}^{2}N_{H}J_{trans}\left(\omega_{F}-\omega_{H}\right)≅const-\frac{2^{1/2}\pi }{45}{\left(\frac{\mu_{0}}{4\pi }\gamma_{H}\gamma_{F}\hbar \right)}^{2}N_{H}{\left(D_{trans}^{CA}\right)}^{-3/2}\sqrt{\omega_{H}-\omega_{F}}$$

(A3) $$\frac{3}{2}{\left(\frac{\mu_{0}}{4\pi }\gamma_{H}\gamma_{F}\hbar \right)}^{2}N_{H}J_{trans}\left(\omega_{F}\right)≅\;const-\frac{3\times 2^{1/2}\pi }{45}{\left(\frac{\mu_{0}}{4\pi }\gamma_{H}\gamma_{F}\hbar \right)}^{2}N_{H}{\left(D_{trans}^{CA}\right)}^{-3/2}\sqrt{\omega_{F}}\;$$

(A4) $$3{\left(\frac{\mu_{0}}{4\pi }\gamma_{H}\gamma_{F}\hbar \right)}^{2}N_{H}J_{trans}\left(\omega_{F}+\omega_{H}\right)≅const-\frac{6\times 2^{1/2}\pi }{45}{\left(\frac{\mu_{0}}{4\pi }\gamma_{H}\gamma_{F}\hbar \right)}^{2}N_{H}{\left(D_{trans}^{CA}\right)}^{-3/2}\sqrt{\omega_{H}+\omega_{F}}$$

where “*const*” denotes frequency independent terms (we denote all such terms in the same way although their values are different).

Thus, one obtains:

(A5) $$R_{1F}^{}\left(\omega_{F}\right)≅const-\frac{2^{1/2}\pi }{45}{\left(\frac{\mu_{0}}{4\pi }\gamma_{H}\gamma_{F}\hbar \right)}^{2}N_{H}{\left(D_{trans}^{CA}\right)}^{-3/2}\left[\sqrt{\omega_{H}-\omega_{F}}+3\sqrt{\omega_{F}}+6\sqrt{\omega_{H}+\omega_{F}}\right]$$

Equation (A5) can be rewritten in the form:

(A6) $$R_{1F}^{}\left(\omega_{F}\right)≅const-\frac{2^{1/2}\pi }{45}{\left(\frac{\mu_{0}}{4\pi }\gamma_{H}\gamma_{F}\hbar \right)}^{2}N_{H}{\left(D_{trans}^{CA}\right)}^{-3/2}\left[{\left(\frac{\omega_{H}-\omega_{F}}{\omega_{F}}\right)}^{1/2}+3+6{\left(\frac{\omega_{H}+\omega_{F}}{\omega_{F}}\right)}^{1/2}\right]\sqrt{\omega_{F}}$$

This leads to Equation (4), which can be simplified to Equation (5) by setting: ${\left(\frac{\omega_{H}-\omega_{F}}{\omega_{F}}\right)}^{1/2}≅0$.

## Appendix B

^19^F spin-lattice relaxation data for a series of ionic liquids: [TEA–C8] [TFSI], [TEA–C10] [TFSI], [TEA–C12] [TFSI], [TEA–C14] [TFSI], and [TEA–C16] [TFSI] plotted versus the squared root of the resonance frequency.

## References

[1] Price W.S. (2008). NMR Diffusometry. Modern Magnetic Resonance.

[2] Price W.S. (2009). NMR Studies of Translational Motion.

[3] Pilar K., Rua A., Suarez S.N., Mallia C., Lai S., Jayakody J., Hatcher J.L., Wishart J.F., Greenbaum S. (2017). Investigation of dynamics in BMIM TFSA ionic liquid through variable temperature and pressure NMR relaxometry and diffusometry. J. Electrochem. Soc..

[4] Kruk D., Masiewicz E., Lotarska S., Markiewicz R., Jurga S. (2021). Correlated Dynamics in Ionic Liquids by Means of NMR Relaxometry: Butyltriethylammonium bis(Trifluoromethanesulfonyl)imide as an Example. Int. J. Mol. Sci..

[5] Carignani E., Juszyńska-Gałązka E., Gałązka M., Forte C., Geppi M., Calucci L. (2021). Translational and rotational diffusion of three glass forming alcohols by 1H field cycling NMR relaxometry. J. Mol. Liq..

[6] Kruk D., Masiewicz E., Lotarska S., Markiewicz R., Jurga S. (2022). Relationship between Translational and Rotational Dynamics of Alkyltriethylammonium-Based Ionic Liquids. Int. J. Mol. Sci..

[7] Kruk D., Meier R., Rachocki A., Korpała A., Singh R.K., Rössler E.A. (2014). Determining diffusion coefficients of ionic liquids by means of field cycling nuclear magnetic resonance relaxometry. J. Chem. Phys..

[8] Seyedlar A.O., Stapf S., Mattea C. (2014). Dynamics of the ionic liquid 1-butyl-3-methylimidazolium bis(trifluoromethylsulphonyl)imide studied by nuclear magnetic resonance dispersion and diffusion. Phys. Chem. Chem. Phys..

[9] Kruk D., Wojciechowski M., Verma Y.L., Chaurasia S.K., Singh R.K. (2017). Dynamical properties of EMIM-SCN confined in a SiO_2_ matrix by means of 1H NMR relaxometry. Phys. Chem. Chem. Phys..

[10] Wencka M., Apih T., Korošec R.C., Jenczyk J., Jarek M., Szutkowski K., Jurga S., Dolinšek J. (2017). Molecular dynamics of 1-ethyl-3-methylimidazolium triflate ionic liquid studied by ^1^H and ^19^F nuclear magnetic resonances. Phys. Chem. Chem. Phys..

[11] Korb J. (2018). Multiscale nuclear magnetic relaxation dispersion of complex liquids in bulk and confinement. Prog. Nucl. Magn. Reson. Spectrosc..

[12] Ordikhani A., Stapf S., Mattea C. (2019). Nuclear magnetic relaxation and diffusion study of the ionic liquids 1-ethyl- and 1-butyl-3-methylimidazolium bis(trifluoromethylsulfonyl)imide confined in porous glass. Magn. Reson. Chem..

[13] Jayakody N.K., Fraenza C.C., Greenbaum S.G., Ashby D., Dunn B.S. (2020). NMR Relaxometry and Diffusometry Analysis of Dynamics in Ionic Liquids and Ionogels for Use in Lithium-Ion Batteries. J. Phys. Chem. B.

[14] Kimmich R., Anoardo E. (2004). Field-Cycling NMR Relaxometry. ChemInform.

[15] Fujara F., Kruk D., Privalov A.F. (2014). Solid state Field-Cycling NMR relaxometry: Instrumental improvements and new applications. Prog. Nucl. Magn. Reson. Spectrosc..

[16] Slichter C.P. (1990). Principles of Magnetic Resonance.

[17] Daniel C. (2005). Introduction: General Theory of Nuclear Relaxation. Adv. Inorg. Chem..

[18] Kowalewski J., Mäler L. (2017). Nuclear Spin Relaxation in Liquids: Theory, Experiments, and Applications.

[19] Hwang L., Freed J.H. (1975). Dynamic effects of pair correlation functions on spin relaxation by translational diffusion in liquids. J. Chem. Phys..

[20] Fries P.H. (2006). Dipolar nuclear spin relaxation in liquids and plane fluids undergoing chemical reactions. Mol. Phys..

[21] Fries P.H., Belorizky E. (1998). Monte Carlo calculation of the intermolecular dipolar spin relaxation in a liquid solution. J. Chem. Phys..

[22] Harmon J.F. (1970). Low frequency spin lattice relaxation in glycerol. Chem. Phys. Lett..

[23] Kruk D., Meier R., Rössler E.A. (2012). Nuclear magnetic resonance relaxometry as a method of measuring translational diffusion coefficients in liquids. Phys. Rev. E Stat. Nonlin. Soft Matter Phys..

[24] Markiewicz R., Klimaszyk A., Jarek M., Taube M., Florczak P., Kempka M., Fojud Z., Jurga S. (2021). Influence of Alkyl Chain Length on Thermal Properties, Structure, and Self-Diffusion Coefficients of Alkyltriethylammonium-Based Ionic Liquids. Int. J. Mol. Sci..

