# Supplementary material for: Relative Cation-Anion Diffusion in Alkyltriethylammonium-Based Ionic Liquids
Source: Int J Mol Sci. 2022 May 26;23(11):5994. doi: 10.3390/ijms23115994 (PMC9181216; doi:10.3390/ijms23115994)
Supplement: Supplementary file 1 [file ijms-23-05994-s001.zip › ijms-1701884-supplementary.pdf]

Supplementary Material

for the article

Relative Cation-Anion Diffusion in Alkyltriethylammonium-based Ionic Liquids

Danuta Kruk[1*], Elzbieta Masiewicz[1], Karol Kolodziejski[1], Roksana Markiewicz[2], Stefan Jurga[2]

[1]Department of Physics and Biophysics, University of Warmia & Mazury in Olsztyn, Oczapowskiego 4, 10-719 Olsztyn, Poland

[2]NanoBioMedical Centre, Adam Mickiewicz University, Poznan, Wszechnicy Piastowskiej 3, 61-614 Poznan, Poland

Examples of ^{19}F magnetisation versus time for a series of ionic liquids: [TEA-C8][TFSI], [TEA-C10][TFSI], [TEA-C12][TFSI], [TEA-C14][TFSI] and [TEA-C16][TFSI], respectively.

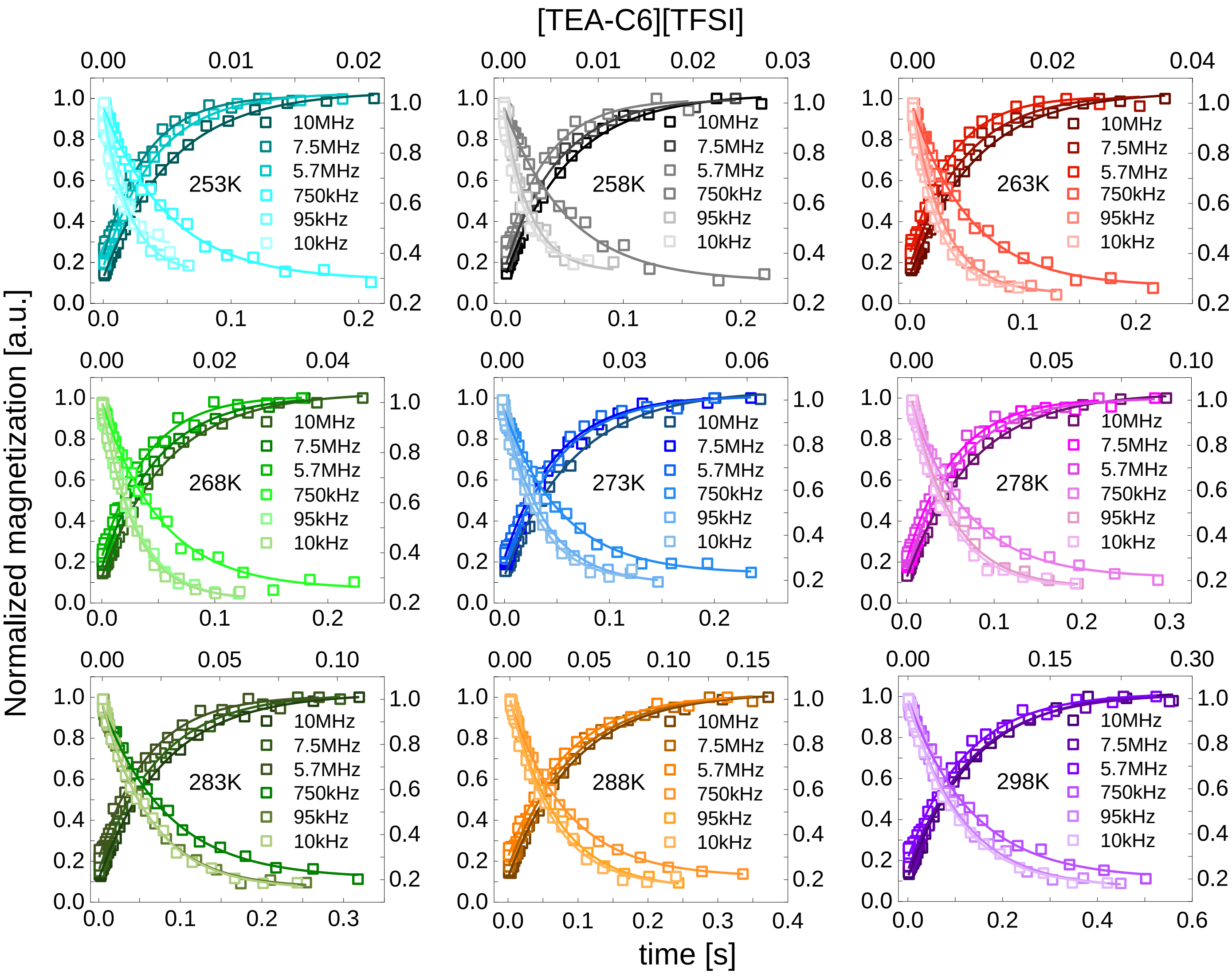

Figure S1. ^{19}F magnetization curves for [TEA-C6] [TFSI]. Solid lines denote single exponential fits. Bottom x and left y – 10MHz, 7.5MHz, 5.7MHz top x right y -750kHz, 95kHz. 10kHz.

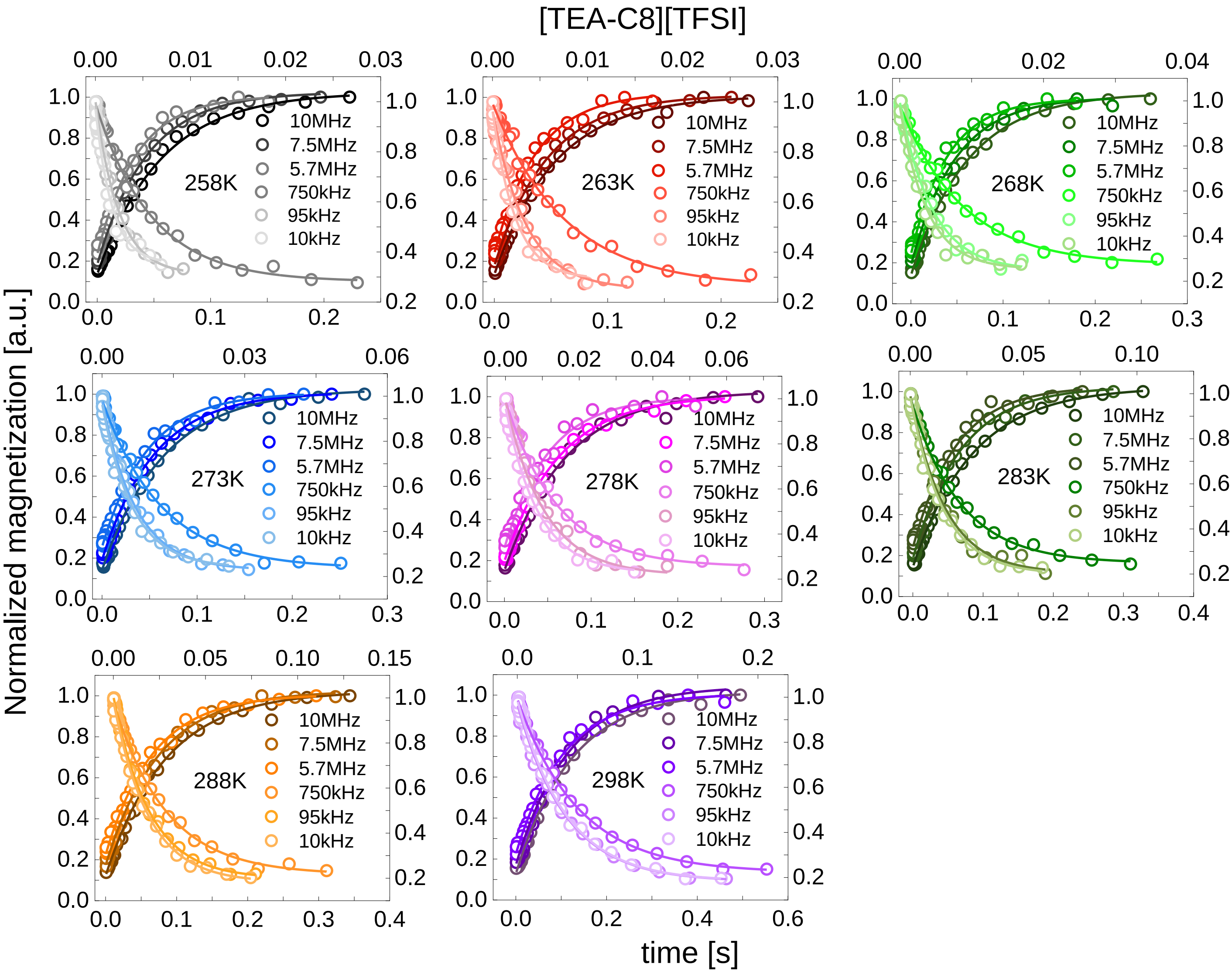

Figure S2. ^{19}F magnetization curves for [TEA-C8] [TFSI]. Solid lines denote single exponential fits. Bottom x and left y – 10MHz, 7.5MHz, 5.7MHz top x right y -750kHz, 95kHz. 10kHz.

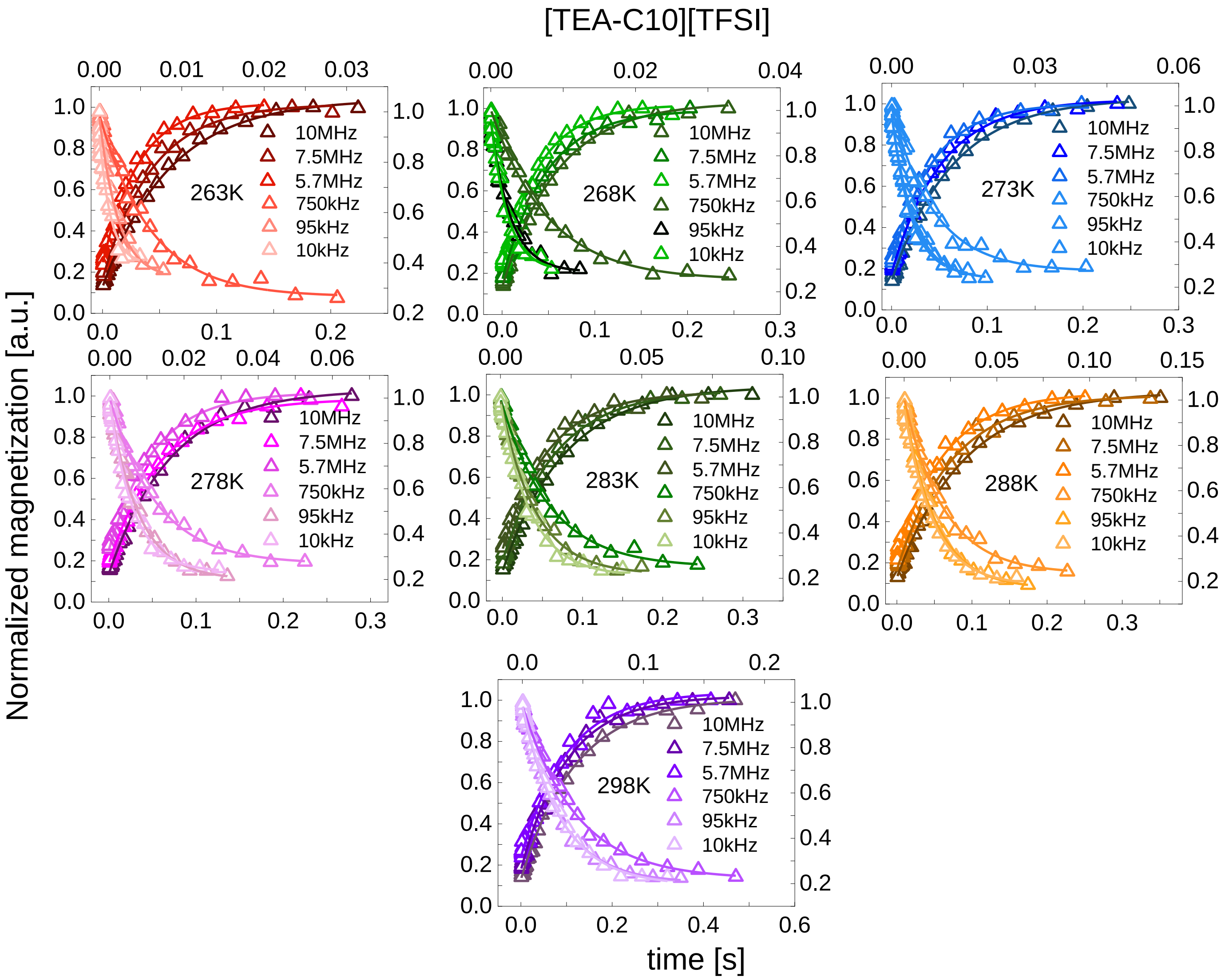

Figure S3.^{19}F magnetization curves for [TEA-C10] [TFSI]. Solid lines denote single exponential fits. Bottom x and left y – 10MHz, 7.5MHz, 5.7MHz top x right y -750kHz, 95kHz. 10kHz.

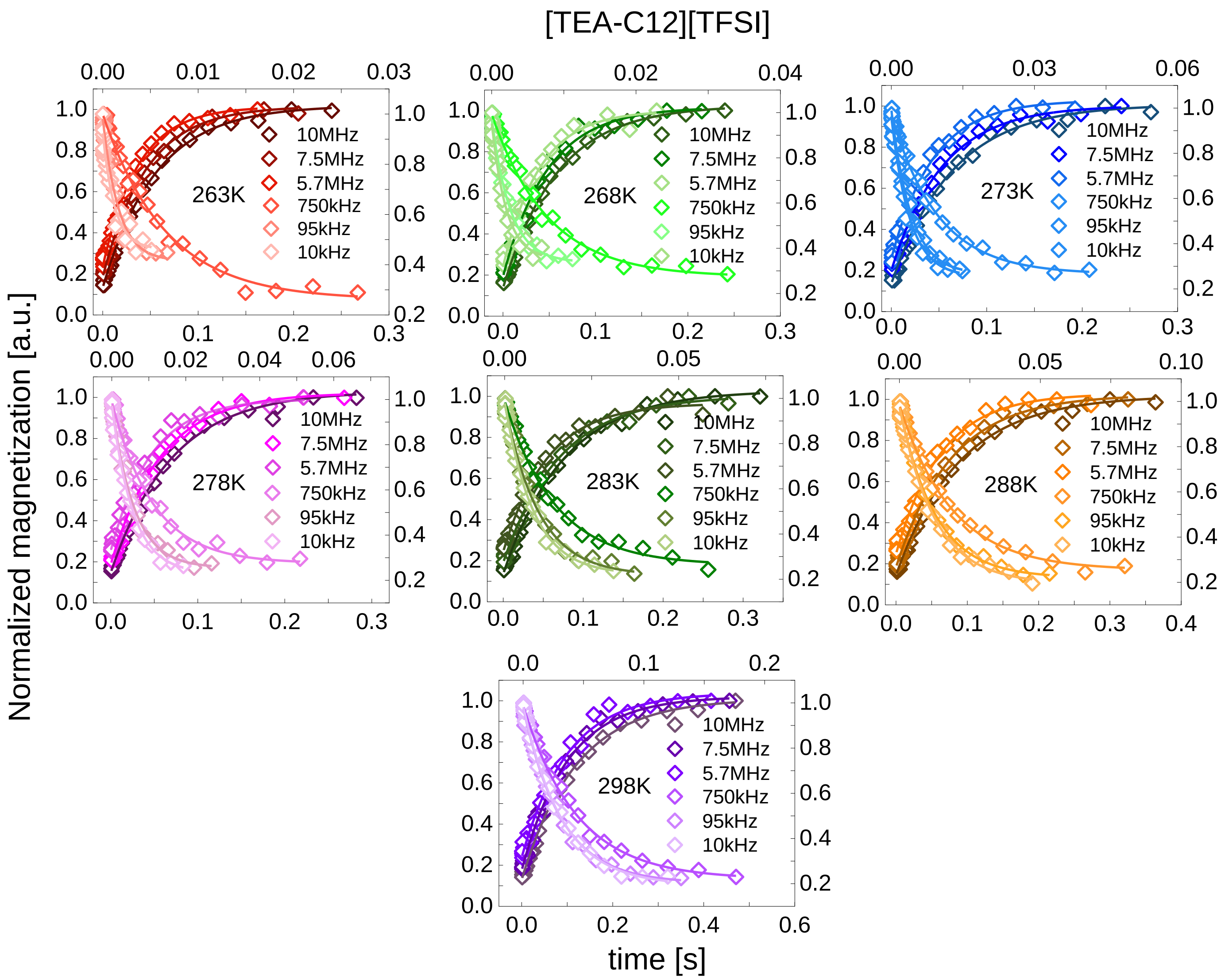

Figure S4. ^{19}F magnetization curves for [TEA-C12] [TFSI]. Solid lines denote single exponential fits. Bottom x and left y – 10MHz, 7.5MHz, 5.7MHz top x right y -750kHz, 95kHz. 10kHz.

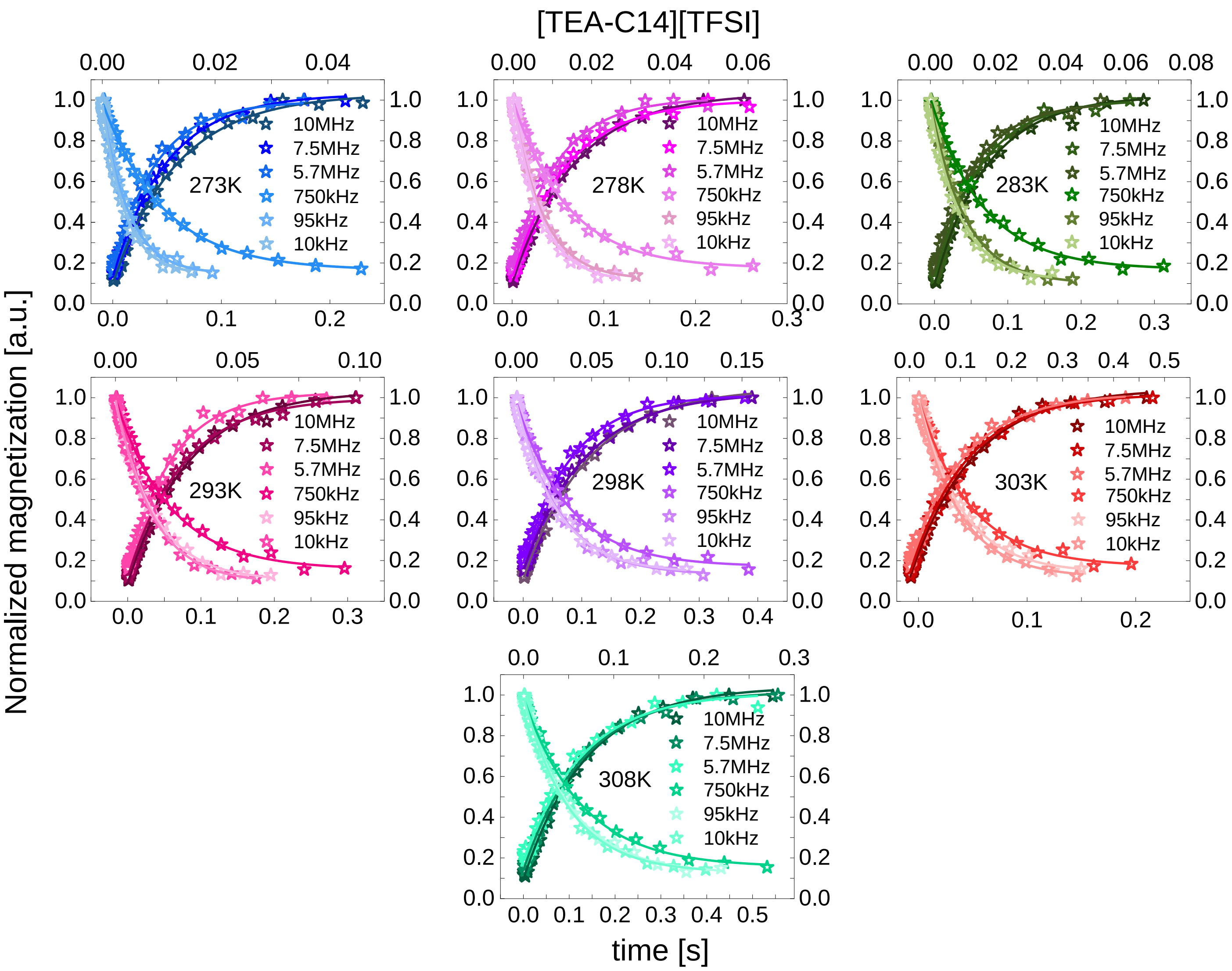

Figure S5. ^{19}F magnetization curves for [TEA-C14] [TFSI]. Solid lines denote single exponential fits. Bottom x and left y – 10MHz, 7.5MHz, 5.7MHz top x right y -750kHz, 95kHz. 10kHz.

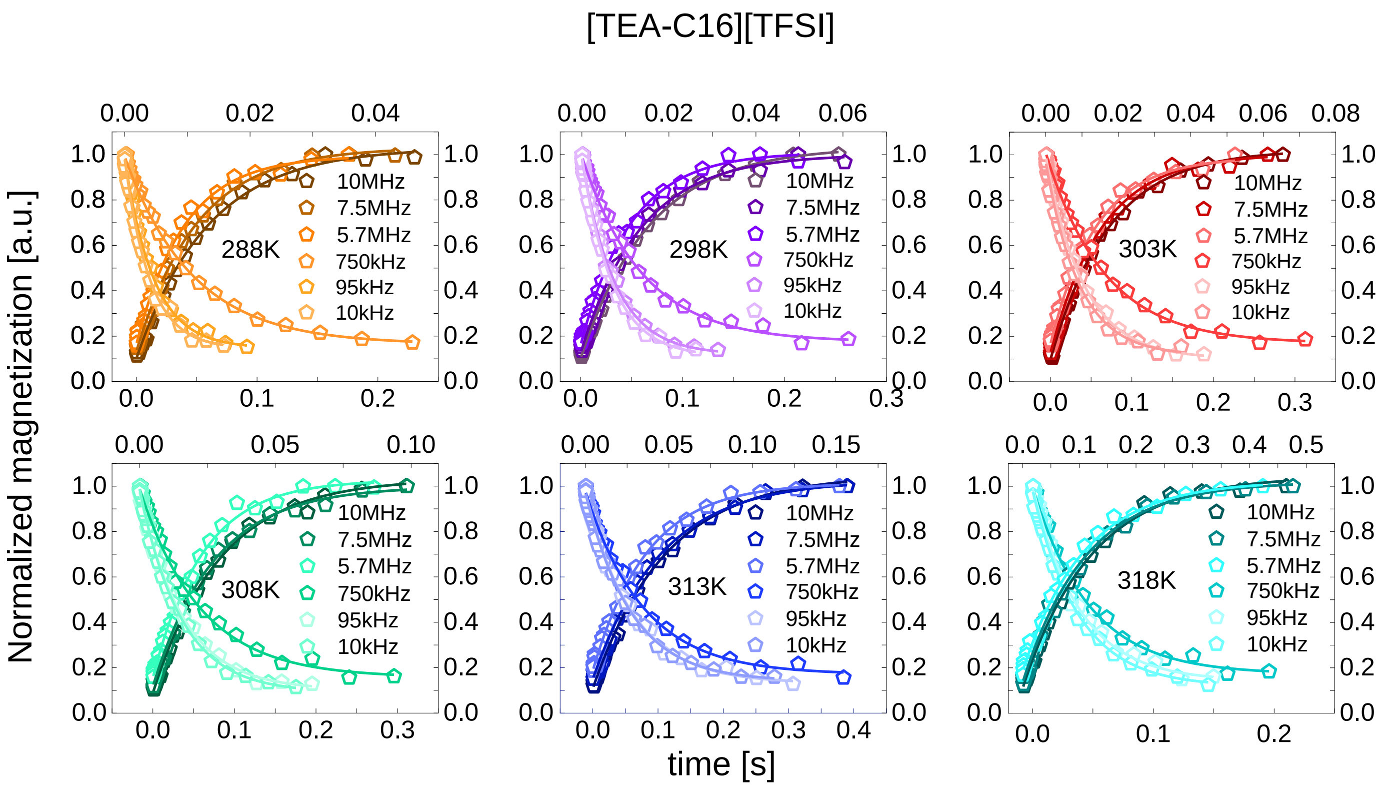

Figure S6. ^{19}F magnetization curves for [TEA-C16] [TFSI]. Solid lines denote single exponential fits. Bottom x and left y – 10MHz, 7.5MHz, 5.7MHz top x right y -750kHz, 95kHz. 10kHz.
